# Responses of Bacterial Communities in Arable Soils in a Rice-Wheat Cropping System to Different Fertilizer Regimes and Sampling Times

**DOI:** 10.1371/journal.pone.0085301

**Published:** 2014-01-20

**Authors:** Jun Zhao, Tian Ni, Yong Li, Wu Xiong, Wei Ran, Biao Shen, Qirong Shen, Ruifu Zhang

**Affiliations:** 1 Key Laboratory of Plant Nutrition and Fertilization in Low-Middle Reaches of the Yangtze River, Ministry of Agriculture, Nanjing, Jiangsu, China; 2 Jiangsu Key Lab and Engineering Center for Solid Organic Waste Utilization, Nanjing Agricultural University, Nanjing, Jiangsu, China; 3 Soil and Fertilizer Technical Guidance Station of Jintan City, Jintan Agricultural and Forestry Bureau, Jintan, Jiangsu, China; 4 Jiangsu Collaborative Innovation Center for Solid Organic Waste Resource Utilization, Nanjing Agricultural University, Nanjing, Jiangsu, China; Wageningen University, Netherlands

## Abstract

Soil physicochemical properties, soil microbial biomass and bacterial community structures in a rice-wheat cropping system subjected to different fertilizer regimes were investigated in two seasons (June and October). All fertilizer regimes increased the soil microbial biomass carbon and nitrogen. Both fertilizer regime and time had a significant effect on soil physicochemical properties and bacterial community structure. The combined application of inorganic fertilizer and manure organic-inorganic fertilizer significantly enhanced the bacterial diversity in both seasons. The bacterial communities across all samples were dominated by *Proteobacteria*, *Acidobacteria* and *Chloroflexi* at the phylum level. Permutational multivariate analysis confirmed that both fertilizer treatment and season were significant factors in the variation of the composition of the bacterial community. Hierarchical cluster analysis based on Bray-Curtis distances further revealed that bacterial communities were separated primarily by season. The effect of fertilizer treatment is significant (*P* = 0.005) and accounts for 7.43% of the total variation in bacterial community. Soil nutrients (e.g., available K, total N, total P and organic matter) rather than pH showed significant correlation with the majority of abundant taxa. In conclusion, both fertilizer treatment and seasonal changes affect soil properties, microbial biomass and bacterial community structure. The application of NPK plus manure organic-inorganic fertilizer may be a sound fertilizer practice for sustainable food production.

## Introduction

Soils are considered to be the most diverse microbial habitats on Earth with respect to species diversity, community size and microbial biomass [1], [2]. Bacteria are the most abundant and diverse group of soil microorganisms [3], playing a vital role in agroecosystems through participation in recycling soil nutrients, maintaining soil structure and promoting plant growth [4], [5]. It has long been recognized that the appropriate community structure, abundant diversity and a high activity of microorganisms are significant factors in maintaining the sustainability and productivity of ecosystems [6]–[9].

Fertilization is an important agricultural practice for improving plant nutrition, increasing soil organic matter and achieving high crop yields, but it can also affect soil microbial abundance, activity and community structure [10]–[13]. Significant differences in soil microbial biomass and microbial diversity have been observed following fertilization [14]–[16]. Organic fertilizers usually increase soil microbial biomass [17], [18], enzyme activities [13], [19] and functional diversity [16], while microbial biomass and enzyme activities are decreased in response to inorganic fertilizers [20]–[22].

Previous studies have indicated that seasonal changes impact soil microbial communities in agroecosystems [23]–[27]. Moreover, temporal variation of conditions is a very common feature of agroecosystems, including temperature, rainfall, plant type, nutrient levels, etc. Hence, we reason that study using single time point sampling cannot tease apart the effect of temporal variation on microbial community structure.

Soil microorganisms respond quickly to environmental changes (e.g., application of fertilizer or herbicide, tillage, crop rotation and seasonal variation), resulting in dynamic changes in microbial biomass, activity, diversity, abundance and composition. Microbial biomass, activity and diversity are effective indicators of soil quality and health [28], [29]. Therefore, understanding the shifts of microbial biomass, community structure and diversity following different agricultural management practices is important for selecting suitable management strategies to improve ecosystem service [30], [31].

The rice-wheat cropping system is one of the main cropping systems for cereal food production in China. Understanding the bacterial community in response to the different fertilizer treatments and seasonal variations will help us disclose the “real” effect of fertilizer regimes and guide us in selecting an appropriate management practice for more stable and sustainable agroecosystem for food production. In the present study, we aimed 1) to study the effect of different fertilizer regimes and seasonal variations on the bacterial community and microbial biomass; 2) to determine whether the fertilizer regime or seasonal change is the major determinant of bacterial community structure; and 3) to determine the contribution of environmental factors on changes in the bacterial community. To address this aim, measurement of biological properties (microbial biomass) and molecular analysis (pyrosequencing) were used to assess the variation in bacterial community.

## Materials and Methods

### Ethic statement

No specific permits were required for the described field studies. The locations are not protected. The field studied did not involve endangered or protected species.

### Field description and experimental design

The field experiment is located in Jintan city, Jiangsu Province, China (31°39′N, 119°28′E, 3 m a.s.l). This region has a northern subtropical monsoon climate, with an average annual temperature of 15.3°C and a mean annual precipitation of 1063.6 mm. The soil type is classified as typical Clay loamy Fe-leachic-gleyic-stagnic anthrosol, with a long-term annual rotation of winter wheat (*Triticum aestivum* L.) and summer rice (*Oryza sativa* L.), which is the typical cropping system in this region. The fertilization experiment has been in operation since 2010, including four replicates of six treatments in a random block design. The treatments were control without fertilizers (CK), chemical fertilizers (NPK), 50% NPK fertilizer plus 400 kg/ha manure (NPKM), 100% NPK fertilizer plus crop straw (NPKS), 50% NPK fertilizer plus 400 kg/ha manure and crop straw (NPKMS) and 30% NPK fertilizer plus 240 kg/ha manure organic-inorganic compound fertilizer (NPKMOI). It is noted that the manure organic-inorganic compound fertilizer were comprised of pig manure compost and suitable amount of chemical fertilizer. Each plot was 5 m × 8 m and received the same levels (except for the control plot) of nitrogen, phosphorus and potassium (N: 240 kg/ha, P_2_O_5_: 120 kg/ha and K_2_O: 100 kg/ha) from fertilizers in each cropping season. All P, K and manure fertilizers were applied as basal fertilizers before planting, whereas N fertilizer (urea) was used both as basal fertilizer before planting and supplementary fertilizer at tillering and panicle stage. The wheat was planted in October and harvested in June, followed by rice, which was planted in June and harvested in October.

### Soil sampling and analysis

Soil samples were taken in summer (June 2012) after the wheat harvest and in autumn (October 2012) after the rice harvest. Ten cores (2.5 cm in diameter) were randomly collected from the plough layer of soil (0–20 cm) in each replicate plot. The cores from each replicate plot were mixed together, pooled in a sterile plastic bag and transported to the laboratory on ice. All the samples were sieved (2 mm), thoroughly homogenized, and divided into three subsamples: one was air-dried for soil characteristic analysis; another was stored at −4°C for the measurement of biological properties; the rest was stored at −80°C for DNA extraction and subsequent molecular analysis. The soil analysis of each sample was performed by the soil testing lab in Qiyang at the red soil experimental station of the Chinese Academy of Agricultural Sciences.

### Microbial biomass

Microbial biomass C (MBC) and biomass N (MBN) were measured using the chloroform fumigation-extraction method [32], [33]. After 24 h fumigation, soils were extracted using 0.5 M K_2_SO_4_ with a 1∶5 ratio for 60 min on a rotary shaker, and C and N were determined on a LiquiTOC element analyzer II (Elementar Analysen-systeme GmbH,Hanau, Germany). Finally, the result was calculated using 0.45 (*k_EC_*) and 0.54 (*k_EN_*) correction factors [34], [35].

### DNA extraction

Three samples (biological replicates) of each fertilizer treatment for each season were used for DNA extraction. Total soil DNA was extracted from 0.5 g of fresh soil using a PowerSoil DNA Isolation Kit (Mo Bio Laboratories Inc., Carlsbad, CA, USA) according to the manufacturer's instructions. The extracted DNA was evaluated on a 1% agarose gel, and the concentration and quality (A_260_/A_280_) of the extracts were determined using a NanoDrop ND-2000 spectrophotometer (NanoDrop, Wilmington, DE, USA). To minimize the DNA extraction bias, three successive DNA extracts of each sample were pooled. The DNA of each sample was diluted 50-fold for further use.

### 16S rRNA gene amplification and 454 pyrosequencing

An aliquot (10 ng) of purified DNA from each sample (one biological replicate) was used as template for amplification. The V1-V3 hypervariable region of the bacterial 16S rRNA was amplified using the primer set 27F: AGAGTTTGATCCTGGCTCAG, with the Roche-454 B sequencing adapter, and 533R: TTACCGCGGCTGCTGGCAC, which contained the Roche-454 A sequencing adapter and a unique, error-correcting 10-bp barcode sequence at the 5′-end of each primer. Each sample was amplified in triplicate in a 20 µl reaction using the following program: 2 min of initial denaturation at 95°C followed by 25 cycles of denaturation (95°C for 30 s), annealing (55°C for 30 s), extension (72°C for 30 s), and a final extension at 72°C for 5 min. The PCR products of each sample were pooled together and purified using a PCR Purification Kit (Axygen Bio, USA). The amplicons of each sample were then combined in equimolar concentrations into a single tube prior to 454 pyrosequencing. Pyrosequencing was performed on a 454 GS-FLX Titanium System (Roche, Switzerland) by Majorbio Bio-pharm Technology Co., Ltd (Shanghai, China).

### Processing of pyrosequencing data

Raw pyrosequencing data were processed using Mothur (version 1.29.2) [36] following the Schloss standard operating procedure [37]. Specially, sequences having a minimum flow length of 360 flows and a maximum flow length of 450 flows were de-noised using the Mothur based re-implementation of the PyroNoise algorithm [38] with the default parameters. De-noised sequences with more than two mismatches to the forward primer sequence, one mismatch to the barcode sequence, containing a homopolymer longer than eight nucleotides, any ambiguous base calls (Ns) or a sequence length shorter than 200 bases were eliminated. Then, the filtered sequences were assigned to soil samples based on the unique 10-bp barcodes. After removing the barcode and primer sequences, the unique sequences were aligned against the Silva 106 database [39]. Through screening, filtering, pre-clustering processes and chimera removal using UCHIME [40], the retained sequences were used to build a distance matrix with a distance threshold of 0.2. Using the average neighbor algorithm with a cutoff of 97% similarity, bacterial sequences were clustered to operational taxonomic units (OTUs) (hereby defined as identified OTUs) and the most abundant sequence in each OTU was selected as the representative sequence. Representative sequences were taxonomically classified using an RDP naïve Bayesian rRNA Classifier [41] with a confidence threshold of 60%. Relative abundance of a given phylogenetic group was set as the number of sequences affiliated with that group divided by the total number of sequences per sample. To correct for sampling effort, we used a randomly selected subset of 4,000 sequences per sample for α-diversity analysis. To increase the sampling intensity, we pooled the sequences belonging to each fertilizer treatment for each season. In addition, to downweight the effects of rare species on ß-diversity analysis, only OTUs with ≥20 counts summed across all samples were retained (hereby defined as retained OTUs).

### Statistical analysis

An OTU-based analysis was performed to calculate the richness, diversity and coverage in our samples with a cutoff of 3% dissimilarity per 4,000 sequences. Richness indices, the Chao1 estimator [42] and the abundance-based coverage estimator (ACE) [43] were calculated to estimate the number of observed OTU that were present in the sampling assemblage. The diversity within each individual sample was estimated using the non-parametric Shannon diversity index [44] and the Simpson diversity index [45]. Good's non-parametric coverage estimator [46] was used to estimate the percentage of the total species that were sequenced in each sample. Rarefaction curves generated using Mothur [36] were also used to compare relative levels of bacterial OTU diversity across all fertilizer treatments in each season. Significant differences were considered when there was no overlap between the 95% confidence intervals.

In all tests, a *P*-value <0.05 was considered statistically significant. All data were tested for normality and homogeneity and the data were log_2_ (x+1)-transformed when necessary to meet the criteria for a normal distribution. A multiple analysis of variance (MANOVA) using PASW Statistics 18 (SPSS Inc.) was used to determine the effects of fertilizer treatment and sampling time on the dependent variables, soil characteristics, bacterial abundance, microbial biomass, relative abundance of abundant taxa and the *α-*diversity indices. If the multivariate *F* was significant, we then proceeded with the individual univariate analysis.

To describe the changes in soil bacterial assemblages among treatments and seasons without biases resulting from differences in sequencing depth, all samples were first rarefied to 3,000 counts using the rrarefy function in the vegan [47] package of R (version 2.15.0) [48] based on the retained OTUs tables. A permutational multivariate analysis of variance [49] was performed to assess the effect of fertilizer regime, sampling time and its interaction on bacterial community structure (abundance of OTUs and phyla) using the adonis function of the R vegan [47] package with 999 permutations. To compare bacterial community structures across all samples based on the OTU composition, hierarchical cluster analysis was carried out using the hclust function with the average linkage algorithm in the stats package of R [48]. Then, the Bray-Curtis and Euclidean distances were used to construct a community dissimilarity matrix and an environmental dissimilarity matrix, respectively. The effects of rare species were downweighted by applying the Hellinger transformation for community data [50]. A Mantel test, using the mantel function in the vegan [47] package of R [48], was used to calculate the correlation between the Bray-Curtis distances of bacterial community and soil characteristics. To visualize the relationship between bacterial communities and environmental factors, a redundancy analysis (RDA) was carried out using the rda function, and the environmental factors were fitted to the ordination plots using the envfit function of the vegan [47] package in R with 999 permutations. Pearson correlations between soil characteristics and abundant phyla (classes) were calculated using PASW Statistics 18 (SPSS Inc.). The BioEnv procedure was used to select the subset of environmental properties best correlated (highest Pearson correlation) with bacterial assemblage dissimilarity, and they were used to construct the soil property matrix for variation partitioning analysis (VPA) in the vegan [47] package of R [48].

### Sequence accession numbers

Sequence data have been deposited in the NCBI Sequence Read Archive (SRA) database with accession number SRA073640.

## Results

### Soil physicochemical characteristics

The soil pH, available N (AN) and available P (AP) were significantly (*P*<0.05) affected by different fertilizer treatments, while organic matter (OM) and total P (TP) were significantly (*P*<0.001) affected by sample time (Table 1). The soil OM content was higher in October (29.3 g/kg) than in June (24.0 g/kg), while the soil TP content was higher in June (1.09 g/kg) than in October (0.68 g/kg). Both treatment and season showed a significant (*P*<0.01) effect on soil total N (TN). In addition, the treatment, sample time and treatment × sample time interaction terms were all significant affected soil available K (AK) content and separate analyses for each season showed that soil AK content was highest in treatment NPKMOI (118.4 mg/kg, 54.6 mg/kg) and lowest in treatment CK (71.4 mg/kg, 42.7 mg/kg) both in June and October (Table S1). No significant differences of soil total K (TK) were observed between different treatments, seasons and the interaction terms.

**Table 1:** Effects of fertilizer regime, sample time and the interaction between them on soil physicochemical characteristics.

	pH	Organic matter (g/kg)	Available N (mg/kg)	Available P (mg/kg)	Available K (mg/kg)	Total N (g/kg)	Total P (g/kg)	Total K (g/kg)
Fertilizer Regime§(FR)								
CK	7.01±0.15 ab	24.9±3.5 a	117.0±7.6 b	8.1±2.9 b	57.0±15.9 c	1.43±0.11 b	0.83±0.24 a	15.3±1.4 a
NPK	6.97±0.14 b	26.4±4.2 a	134.2±9.7 a	13.8±4.3 ab	64.5±21.2 bc	1.52±0.10 ab	0.87±0.25 a	15.3±1.2 a
NPKM	7.11±0.19 ab	26.6±3.4 a	136.6±7.5 a	14.5±1.8 ab	66.1±21.7 bc	1.59±0.09 a	0.90±0.24 a	16.1±1.5 a
NPKS	7.13±0.07 ab	27.3±4.5 a	137.5±7.0 a	15.2±3.5 a	76.3±28.3 ab	1.62±0.11 a	0.89±0.21 a	15.4±2.2 a
NPKMS	7.27±0.19 a	27.7±4.2 a	135.0±6.5 a	16.6±4.4 a	70.7±23.9 b	1.64±0.17 a	0.92±0.22 a	16.0±1.7 a
NPKMOI	7.07±0.16 ab	27.3±3.6 a	138.5±5.9 a	15.1±3.1 a	86.5±36.0 a	1.59±0.07 a	0.89±0.22 a	15.6±1.7 a
Sample Time (ST)								
June	7.04±0.17 a	24.0±2.2 b	131.2±10.2 a	12.4±3.1 a	91.9±17.1 a	1.50±0.10 b	1.09±0.07 a	15.2±1.0 a
October	7.12±0.18 a	29.3±3.0 a	137.7±8.0 a	13.8±4.1 a	48.5±5.4 b	1.63±0.12 a	0.68±0.05 b	16.0±1.9 a
ANOVA *P*-values								
FR	0.021	NS	< 0.001	0.009	< 0.001	0.003	NS	NS
ST	NS	< 0.001	NS	NS	< 0.001	< 0.001	< 0.001	NS
FR × ST	NS	NS	NS	NS	0.008	NS	NS	NS

Values are means ± standard deviation (n = 6 or n = 18).

NS: not significant (*P*>0.05).

Fertilizer regimes: CK: control without fertilizers; NPK: chemical fertilizers; NPKM: 50% NPK fertilizer plus 400 kg/ha manure; NPKS: 100% NPK fertilizer plus crop straw; NPKMS: 50% NPK fertilizer plus 400 kg/ha manure and crop straw; NPKMOI: 30% NPK fertilizer plus 240 kg/ha manure organic-inorganic compound fertilizer.

Means followed by the same letter for a given factor are not significantly different (*P*<0.05; Turkey's HSD test where there are more than two treatment levels).

### Microbial biomass

No significant differences (*P*<0.05) of soil MBC and MBN were observed between the fertilizer treatment × sample time interactions (Table 2). The soil MBC changed significantly (*P*<0.001) between different treatments, being highest in treatment NPKMOI (541 mg/kg, 402 mg/kg) and lowest in treatment CK (359 mg/kg, 234 mg/kg) both in June and October (Table S2). Soil MBC also varied significantly (*P*<0.001) over time, being higher in June (479 mg/kg dry soil) compared with October (325 mg/kg dry soil). The greatest differences (*P*<0.001) in MBN were between different treatments, with season having a smaller but significant (*P* = 0.043) effect. The soil MBN was higher in June (32.8 mg/kg dry soil) than in October (29.7 mg/kg dry soil). The highest and lowest MBN were also observed in treatment NPKMOI (42 mg/kg, 36 mg/kg) and CK (21 mg/kg, 19 mg/kg) both in June and October, respectively (Table S2).

**Table 2:** Effects of fertilizer regime, sample time and the interaction between them on soil microbial biomass carbon and nitrogen.

	Microbial biomass C (mg/kg)	Microbial biomass N(mg/kg)
Fertilizer Regime§ (FR)		
CK	310.76±89.51 b	20.29±6.45 d
NPK	387.75±88.13 ab	28.36±2.49 c
NPKM	428.73±100.23 a	33.71±7.24 abc
NPKS	398.00±117.64 ab	30.22±3.07 bc
NPKMS	447.68±103.02 a	36.00±5.48 ab
NPKMOI	471.20±87.32 a	38.85±4.93 a
Sample Time (ST)		
June	479.04±96.25 a	32.77±8.18 a
October	325.04±93.96 b	29.71±7.29 b
ANOVA *P*-values		
FR	< 0.001	< 0.001
ST	< 0.001	0.043
FR × ST	NS	NS

Values are means ± standard deviation (n = 8 or n = 24).

NS: not significant (*P*>0.05).

Means followed by the same letter for a given factor are not significantly different (*P*<0.05; Turkey's HSD test where there are more than two treatment levels).

Fertilizer regimes as described in Table 1.

### Bacterial community composition

Across all soil samples, we obtained 262,299 quality sequences in total, with 4,366–12,538 sequences per sample (mean  = 7,284) and were able to classify 86.5% of those sequences at the phylum level. The dominant phyla across all samples were *Proteobacteria*, *Acidobacteria*, *Chloroflexi*, *Bacteroidetes*, *Actinobacteria*, *Gemmatimonadetes*, *Verrucomicrobia* and *Nitrospira* (>1%), accounting for more than 74% of the bacterial sequences from each of the soils (Table S3; Table S4). In addition, *WS3*, *Firmicutes*, *Armatimonadetes*, *TM7*, *Planctomycetes* and *Chlorobi* were in low abundance, and 10 other rare phyla were identified (Table S3).

No significant (*P*<0.05) interaction between treatment and time was observed for abundant phyla (>1%) (Table S4), while the phylum distribution fluctuated under different fertilizer treatments and seasons. *Chloroflexi*, *Verrucomicrobia* and *Nitrospira* were significantly (*P*<0.001) affected by sample time, while only phylum *Gemmatimonadetes* was significantly (*P*<0.05) affected by fertilizer treatments. In addition, phyla *Acidobacteria*, *Bacteroidetes* and were affected by both treatment and time. No significant differences of *Proteobacteria* and *Actinobacteria* were observed between different treatments, seasons and the interaction terms.

The relative abundance of abundant bacterial classes (subgroups) demonstrating a significant difference under different fertilizer treatments and times is outlined in Table 3. Likewise, no significant (*P*<0.05) interactions between treatment and time were observed for abundant classes (subgroups). Specifically, we observed that both treatment and time had a significant (*P*<0.05) effect on the *α-Proteobacteria*, *ß-Proteobacteria*, and *γ-Proteobacteria*, while the phylum *Proteobacteria* was not affected by treatment, time or the interaction term. Moreover, the relative abundances of different proteobacterial classes were different in response to different fertilizer regimes or seasons. Classes *ß-Proteobacteria* and γ-*Proteobacteria* were higher in June, while the *α-Proteobacteria* was higher in October (5.7%) than in June (4.5%). The treatment NPKMOI cultures higher relative abundances of *α-Proteobacteria* and γ-*Proteobacteria*, while the *ß-Proteobacteria* was higher in treatment NPK, NPKM, NPKS. Within the *Acidobacteria*, subgroup 3 was only affected by time, while subgroups 4 and 6 were affected by treatment. This is not consistent with the phylum *Acidobacteria*, which is affected by both treatment and time. We also observed that the relative abundance of *Acidobacteria* subgroup 3 was higher in October than in June, which was opposite in the phylum level. In addition, *Anaerolineae* and *Sphingobacteria* were the most abundant subgroups in phyla *Chloroflexi* and *Bacteroidetes*, occupying the majority relative abundance in each phylum, respectively. So the effects of the treatment, time and interaction term were consistent at the phylum and class level. However, we also found that the phylum *Bacteroidetes* was significant (*P*<0.05) affected by both treatment and time, while the subgroups *Sphingobacteria* was only affected by time.

**Table 3:** Effect of fertilizer regime, sample time and the interaction between them on the relative abundance (%) of abundant bacterial classes (subgroups) £ (relative abundance >1%).

Phylum	*Proteobaceria*			*Acidobacteria*				*Chloroflexi*	*Bacteroidetes*
Class (subgroups)	*α-proteobacteria*	*ß-proteobacteria*	*γ-proteobacteria*	6	4	7	3	*Anaerolineae*	*Sphingobacteria*
Fertilizer Regime § (FR)									
CK	4.8±0.9 ab	13.4±0.9 ab	4.3±0.6 c	6.1±0.4 a	3.4±1.2 a	2.2±0.5 a	1.3±0.2 a	10.3±2.6 a	4.6±1.6 a
NPK	4.5±1.1 b	13.6±1.7 a	5.3±0.9 bc	5.1±0.9 ab	3.0±1.2 a	1.8±0.4 ab	1.5±0.5 a	11.3±3.0 a	5.2±1.4 a
NPKM	4.2±1.2 b	14.0±1.2 a	5.8±1.0 b	5.9±0.6 a	3.3±1.0 a	2.1±0.2 a	1.5±0.3 a	9.8±1.8 a	5.4±1.4 a
NPKS	4.0±1.4 b	14.8±0.7 a	5.8±0.9 b	5.9±0.6 a	3.3±0.9 a	2.1±0.2 a	1.2±0.4 a	9.0±1.9 a	5.2±1.6 a
NPKMS	5.9±2.1 ab	13.2±1.0 ab	5.2±0.9 bc	5.4±0.6 a	2.8±1.1 ab	1.8±0.3 ab	1.7±0.4 a	10.8±3.6 a	5.0±1.7 a
NPKMOI	7.1±1.6 a	11.7±1.0 b	7.6±1.4 a	4.0±1.1 b	2.1±0.7 b	1.5±0.2 b	1.8±0.4 a	9.7±3.1 a	5.1±2.1 a
Sample Time (ST)									
June	4.5±1.9 b	13.9±1.2 a	6.3±1.3 a	5.5±0.8 a	3.8±0.7 a	2.0±0.4 a	1.4±0.3 b	8.3±1.3 b	6.6±0.6 a
October	5.7±1.4 a	13.0±1.4 b	5.0±1.1 b	5.2±1.1 a	2.1±0.5 b	1.9±0.3 a	1.7±0.5 a	12.0±2.3 a	3.7±0.5 b
ANOVA *P*-values									
FR	0.005	0.001	< 0.001	< 0.001	< 0.001	0.005	NS	NS	NS
ST	0.019	0.013	< 0.001	NS	< 0.001	NS	0.026	< 0.001	<0.001
FR × ST	NS	NS	NS	NS	NS	NS	NS	NS	NS

Values are means ± standard deviation (n = 6 or n = 18).

NS: no significant (*P*>0.05).

Means followed by the same letter for a given factor are not significantly different (*P*<0.05; Turkey's HSD test where there are more than two treatment levels).

*Actinobaceria*, *Gemmatimonadetes*, *Nitrospira* were not included in this analysis as they had only one class.

Fertilizer regimes as described in Table 1.

### Bacterial α-diversity

Bacterial diversity and richness in individual samples under different fertilizer regimes of both seasons were calculated based on 4,000 sequences (Table S5). Statistically significant differences in richness and diversity (*P*≤0.01) were observed only at the season level for observed OTUs, coverage, ACE, Chao1 and Simpson but not for Shannon. The number of OTUs, ACE, Chao1 and Simpson were higher in October in comparison to June, while the percentage showed the opposition pattern, being higher in June (80%) than October (76%). Although the fertilizer regimes had no significant effect on the bacterial diversity and richness, a higher number of observed OTUs, ACE, Chao1 and Shannon were observed with treatment NPKMS and NPKMOI in both seasons, indicating that rational fertilization can help increase the biodiversity in agricultural soils. Further analysis of rarefaction curves demonstrated that the number of observed OTUs for treatment NPKMS and NPKMOI were significantly higher than for other fertilizer treatments in June and treatment NPKMOI had a highest observed OTU numbers in October (Fig. S1), calculated based on 10,000 sequences randomly selected from the pooled sequences of each treatment in each season.

### Bacterial community structure

**Figure 1:**
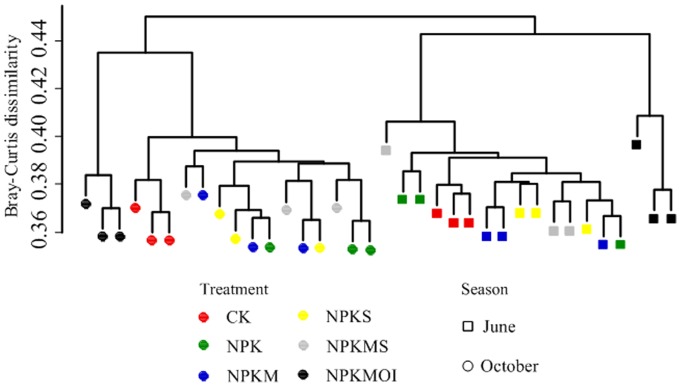
Hierarchical cluster dendrogram of bacterial communities. Pairwise Bray-Curtis dissimilarity of samples collected from six fertilizer treatments in June (square) and October (circle). OTU counts were rarefied to 3,000 counts per sample, and only OTUs ≥20 counts summed across all samples were included in the analysis with the Hellinger-transformation. Symbols of fertilizer regimes were as described in Table 1.

A permutational multivariate analysis of variance confirmed that treatment and time were significant factors of variation for the composition of bacterial community in terms of both the relative abundance of OTUs and relative abundance of phylum (Table 4). No significant interaction between treatment and time were observed. Hierarchical cluster analysis of the similarity of bacterial community further confirmed that sampling time is the major determinant of bacterial community structure (Fig. 1). We also found that the treatment NPKMOI was separated from other treatments in both seasons, indicating this fertilizer treatment may have the greatest effect on the bacterial community.

**Table 4:** Permutational multivariate analyses of variance of the Bray dissimilarity between bacterial communities.

Source	df	Abundance of OTUs		Abundance of phylum	
		Sums of sqs	Pseudo-F	Sums of sqs	Pseudo-F
Fertilizer regime (FR)	5	0.6079	1.7276***	0.014495	2.6929**
Sample time (ST)	1	0.40962	5.8206***	0.033321	30.9527***
FR × ST	5	0.38064	1.0818	0.006292	1.1690
Residuals	24	1.68897		0.025836	

indicate significant correlations (*P*<0.05); ** indicate significant correlations (*P*<0.01); *** indicate significant correlations (*P*<0.001).

**Figure 2:**
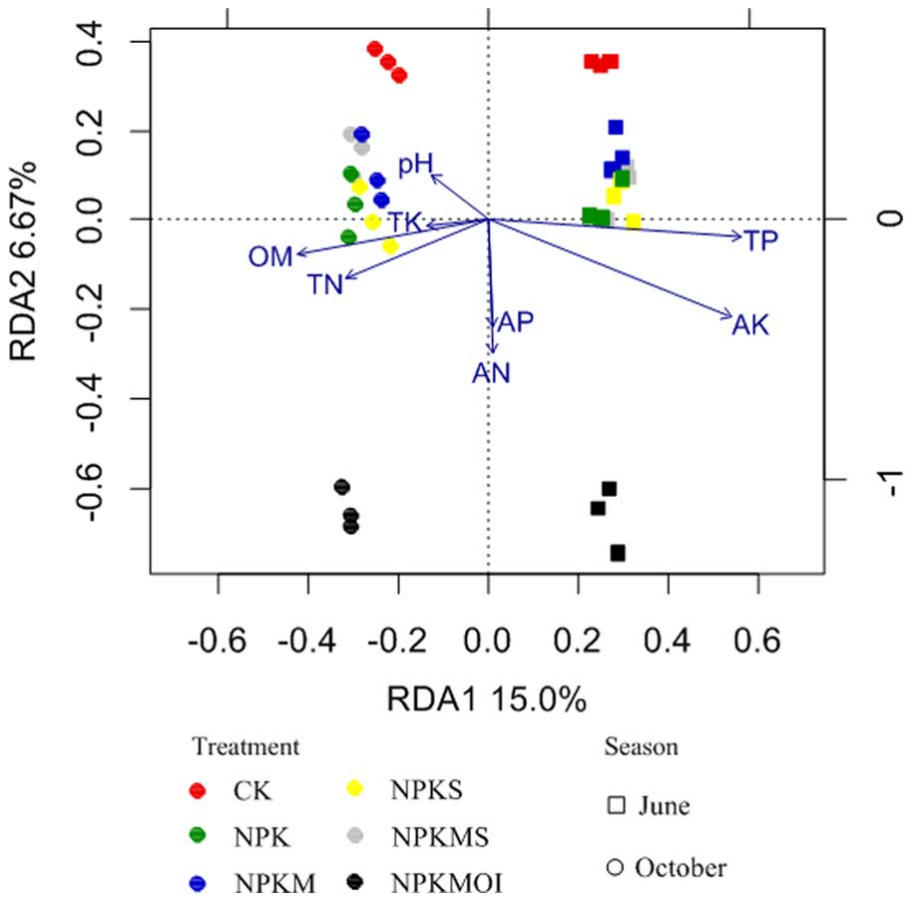
Redundancy analysis of soil bacterial communities and soil characteristics for individual samples. Samples from different fertilizer regimes both in June (square) and October (circle) were marked by different colors. Symbols of fertilizer regimes were as described in Table 1.

The Mantel test showed significant (*r* = 0.44, *P*<0.001) correlation between bacterial community and soil properties. The RDA (redundancy analysis) biplot clearly showed the relationship between soil characteristics and bacterial community structure (Fig. 2). The first two axes of RDA can explain 15.0% and 6.67% of the total variation in the data. The June bacterial communities were grouped separately from the October bacterial communities along the first axis, with the June bacterial communities more relating to higher contents of soil AK and TP, while the October bacterial communities were associated with higher contents of soil pH, OM, TK and TN. Along the second RDA axis, the bacterial communities of treatment NPKMOI and CK were separated from the other fertilizer treatments in both seasons. The treatments NPK, NPKM, NPKS and NPKMS were grouped together in both seasons, indicating that they may have approximately the same effect on the bacterial assembles. The effect of soil properties on bacterial community is shown by the direction and the length of the vectors. The results showed that the soil OM, TP, AK and TN had a significant (*P*<0.01) correlation between each variable and the ordination scores.

In addition, we used Pearson's correlation coefficient to evaluate relationships between abundant phyla and classes (subgroups) (Table 5). Most of the abundant phyla or classes were positively or negatively correlated with soil OM, AK, TN and TP, while only phylum *Verrucomicrobia* showed a significant positive correlation with soil pH and TN. We also found that soil AP content had no significant correlation with any of the abundant taxa. The relative abundances of phylum *Proteobacteria* and class *γ-Proteobacteria* were positively correlated with soil AN and AK, while the *γ-Proteobacteria* was positively correlated with soil TP. The abundance of *Acidobacteria* was negatively correlated with soil OM, AN, TN and TP while positively correlated with soil AK content.

**Table 5:** Pearson's correlation coefficient between soil characteristics and abundant phyla (classes).

Phylum (class)	Correlation							
	pH	Organic matter	Available N	Available P	Available K	Total N	Total P	Total K
*Proteobacteria*	0.203	0.091	**0.393** *	0.221	0.479^**^	0.178	0.327	0.100
*Alphaproteobacteria*	0.159	0.220	0.042	0.214	−0.156	0.116	−0.282	−0.003
*Betaproteobacteria*	0.096	−0.103	0.066	−0.083	0.197	−0.041	0.282	0.058
*Deltaproteobacteria*	−0.046	−0.023	0.056	−0.160	0.101	−0.032	0.187	−0.018
*Gammaproteobacteria*	−0.079	−0.152	**0.407** *	0.269	0.680^**^	0.064	0.472^**^	0.030
*Acidobacteria*	−0.304	−0.451^**^	−0.455^**^	−0.209	**0.420** *	−**0.418** *	−0.541^**^	−0.237
*Acidobacteria_subgroups6*	0.060	−0.146	−0.127	−0.182	0.025	−0.172	0.151	−0.060
*Acidobacteria_subgroups4*	−0.212	−0.673^**^	−0.107	−0.105	0.644^**^	−0.569^**^	0.787^**^	−0.262
*Acidobacteria_subgroups7*	−0.155	−0.130	−0.277	−0.193	−0.070	−0.159	0.079	0.066
*Acidobacteria_subgroups3*	−0.042	0.261	−0.093	0.036	−0.292	0.288	−**0.343** *	−0.063
*Chloroflexi*	0.035	0.452^**^	−0.145	−0.153	−0.753^**^	**0.342** *	−0.720^**^	0.019
*Bacteroidetes*	−0.138	−0.639^**^	0.160	0.175	0.836^**^	−0.461^**^	0.898^**^	−0.220
*Actinobacteria*	0.093	−0.179	0.118	0.197	0.170	−0.201	0.046	−0.037
*Gemmatimonadetes*	−0.272	0.120	0.102	0.108	−0.240	0.041	−0.248	0.021
*Verrucomicrobia*	**0.354** *	**0.368** *	−0.095	0.120	−0.580^**^	0.261	−0.591^**^	0.550^**^
*Nitrospira*	−0.152	−0.568^**^	−0.011	0.073	0.782^**^	−0.447^**^	0.838^**^	−0.202

Values in boldface type indicate significant correlations with *P* values indicated in superscript.

indicate significant correlations (*P*<0.05); ^**^ indicate significant correlations (*P*<0.01).

### Linking the bacterial communities to soil characteristics, sample time and fertilizer regime

**Figure 3:**
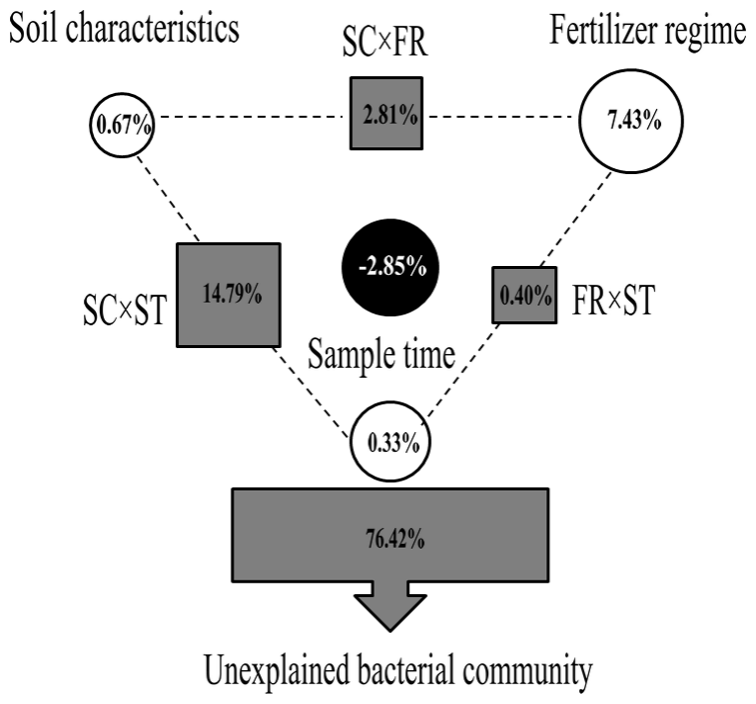
Variation partitioning analysis of the bacterial communities. Effects of soil characteristics (SC), fertilizer regime (FR), sample time (ST) and the interactions between them on the bacterial community structure. Circles on the edges of the triangle show the percentage of variation explained by each factor alone. The percentage of variation explained by interactions between two or three of the factors is shown as squares on the sides and as circle in the center of the triangle. The unexplained variation is depicted in square on the bottom.

Variance partitioning analysis (VPA) was used to determine the relative contributions of sample time, fertilizer regime and soil characteristics to the bacterial communities. A subset of environmental parameters (OM, TP, AK and TK) was selected by the BioEnv procedure, which provides the highest Pearson correlation with bacterial communities. The variation in bacterial community structure was partitioned among soil characteristics, sample time and fertilizer regime, as well as interactions between them. These variables explained 23.58% of the observed variation, leaving 76.42% of the variation unexplained (Fig. 3). Soil characteristics and sample time explained small portions of the observed variation, which accounted for 0.67% (*P* = 0.18) and 0.33% (*P* = 0.255), respectively, while the fertilizer regime could explain 7.43% (*P* = 0.005) of the total variation. The variation was mostly explained by interactions between soil characteristics and sample time, which accounted for 14.79%. The interactions between soil characteristics and fertilizer regime and sample time and fertilizer regime accounted for 2.81% and 0.40% of the variation, respectively. Thus, the fertilizer regimes are important factors in explaining the shifts in bacterial community structures.

## Discussion

The present study attempts to assess the effect of fertilizer regime and sampling time on bacterial communities in a rice-wheat cropping system. The results demonstrate that both fertilizer treatment and season were found to impact soil chemical parameters, microbial biomass, bacterial abundance and bacterial community structure. Soil pH was lower in treatment NPK when compared to other fertilizer treatments and CK (Table 1). This might be due to the application of chemical fertilizer alone, which could acidify the soil [51], while the application of crop straw or pig manure in combination with chemical fertilizer can stabilize the soil pH [52]. We observed that soil available nutrients (AN, AP and AK) were significantly altered by fertilizer treatment in comparison to CK, with the treatment NPKMOI having the highest concentration of soil available nutrients. Application of organic-inorganic compound fertilizer can slowly release of nutrients into the soil, promote plant growth and increase crop yields [53]. However, the treatment NPKMOI did not show any significant differences in soil AN and AP contents compared to other fertilizer regimes. We also did not observe any significant difference in soil organic matter content not only between different fertilizer treatments but also between treatments and CK. The results of soil characteristics indicated that different fertilizer regimes gradually but not dramatically changed soil attributes.

Soil MBC and MBN were found to be higher in June than that in October, suggesting that the soil MBC and MBN contents may be affected by the atmospheric temperature. This result is consistent with previous studies [10], [54]. Kikuchi et al. [55] and Noll et al. [56] found that bacterial community structure and diversity in a paddy soil can be affected by flooded/upland conditions and oxygen gradient. So we consider that the flooding period during rice cultivation in our study is also an important factor in soil microbial biomass shifting. We also found that the application of fertilizer can markedly affect the soil MBC and MBN both in June and October when compared with CK. This suggests the different fertilizers can also activate the microorganisms in soil.

In this study, we applied 454-pyrosequencing of the 16S rRNA gene to characterize the bacterial community under different fertilizer treatments and sampling times. The sequence analysis reveals that phyla *Proteobacteria*, *Acidobacteria*, *Chloroflexi* and *Bacteroidetes* were the most abundant phyla in all of the samples. This result roughly corresponded with previous studies demonstrating that *Proteobacteria*, *Acidobacteria* and *Bacteroidetes* are dominant soil bacterial taxa using sequencing of 16S rRNA gene clone libraries or pyrosequencing [57], [58], while the phylum *Chloroflexi* showed an unexpectedly high relative abundance in our study. Specifically, a higher average abundance of *Chloroflexi* was observed in October (12.37%) than in June (8.66%) (Table S3), which suggests that the practice of periodic flooding (resulting in an anaerobic environment) in the rice season can significantly impact this facultative anaerobic phylum. Significant differences in soil bacterial community composition were observed both among fertilizer treatments and between sampling times. In particular, the highest relative abundance of *α-Proteobacter*ia and *γ-Proteobacteria,* but the lowest of *ß-Proteobacteria,* was observed in treatment NPKMOI in both seasons. *ß-Proteobacteria* are considered as copiotrophic bacteria and always flourish in soils with large amounts of available nutrients [59], while the available nutrients in the treatment NPKMOI are higher than other fertilizer treatments and CK. Therefore, lower relative abundance of *ß-Proteobacteria* in the treatment NPKMOI could be due to other factors. Interestingly, the phylum and its classes or subgroups had different patterns in response to the different treatments and seasons. For instance, classes *α-Proteobacter*ia, *ß-Proteobacteria* and *γ-Proteobacteria* were all affected by both treatment and season, while the phylum *Proteobacteria* was not affected by treatment, season or the interaction between fertilizer and season. In contrast, the phylum *Acidobacteria* was affected by both treatment and season, subgroups 6, 7 and 3 were only affected by treatment or season. This indicates that finer taxa may have more sensitive responses to different treatments and seasons due to its own characteristics.

Many studies have shown that environmental factors determine the soil microbial community [5], [60], [61], especially the soil pH, which has been demonstrated in several studies to be the strongest factor shaping microbial community structures [60], [62]–[64]. Chu et al. [65] and Rousk et al. [60] have reported that the relative abundances of *α-Proteobacter*ia and *γ-Proteobacteria* increase with higher soil pH, but we did not find the same trend, which may be due to the mild fluctuations (range from 6.86 to 7.37 in all samples) in the soil pH in our study. Jones et al. [66] reported that relative abundances of Acidobacterial subgroups 4, 6 and 7 were positively correlated with soil pH, which appears inconsistent with our results, being higher in treatment CK than that in treatment NPKMOI. The soil pH values varied significantly from 3 to 9 in those studies allowing insight into the relationship between pH and soil bacterial communities besides that the soil factors assessed in this study were not measured in the studies of Chu et al., [65], Roush et al., [60] and Jones et al., [66]. Thus, we hypothesize that there are important factors other than pH in shaping soil bacterial communities, which was similar with Navarrete et al., [67], demonstrating that abiotic soil factors (not only related to soil acidity) such as Al, Ca, Mg, Mn and B can also drive the acidobacterial populations. Further Pearson correlation analysis showed that only phylum *Verrucomicrobia* had a small correlation with soil pH. In contrast, we found that most of the proportions of abundant phyla and classes (subgroups) were highly correlated with soil OM, AK, TN and TP contents (Table 5). Lauber et al. [62] considered that the soil pH may not directly alter bacterial community structure but may instead function as a variable that provides an integrated index of soil conditions. In fact, there are a number of soil characteristics (e.g., nutrient availability, organic C characteristics, soil moisture regimen and salinity) that are often directly or indirectly related to soil pH [68], and these factors may drive the observed changes in community composition as the hydrogen ion concentration varies by many orders of magnitude across the soils [60], [62]–[64]. However, the soil pH may not represent an integrating variable in our study due to the mild variation of soil pH, which suggests that soil characteristics such as soil nutrients, organic matter, soil moisture, etc. rather than soil pH can shape the bacterial communities by themselves. In addition, Nacke et al. [5] found that the phylum *Actinobacteria* was positively correlated with TN and class *δ-Proteobacteria* was negatively correlated with organic C. However, these two bacterial taxa had no significant correlation with any soil properties in our study (Table 5). Hence, we hypothesize that soil nutrients can also drive the bacterial community composition and that the correlation patterns are varied in different soils.

Soil microbial diversity is considered to be critical to the integrity, function and long-term sustainability of soil ecosystems [69]. Moreover, greater biodiversity in soil can lead to a more stable system and enhance the combination of vital microbial functions and processes [9]. In the present study, higher biodiversity was observed in treatment NPKMOI at both seasons, indicating that combined application of chemical fertilizer and organic-inorganic compound fertilizer can maintain a higher biodiversity. It may result in a more stable agroecosystem and may contribute to sustainable crop production.

Considerable temporal variation in soil bacterial communities has been previously been reported for agricultural soils [24], [26], [27]. In the present study, the greatest community variation was also found to be temporal (Fig. 1). All the soil samples showed temporal clustering and higher similarity was observed within each season. Bremer et al. [70] considered that plant presence may result in seasonal dynamics of bacterial communities. In our study, we collected soil samples after crop harvest to avoid this bias. However, we also found that within each season, the bacterial community structure of treatment NPKMOI differed from other treatments or CK (Fig. 1 and Fig. 2), indicating that fertilization is also an important factor in shaping bacterial community. This was verified by the permutational multivariate analysis of variance of the bacterial community (Table 4). In addition, soil properties were also significantly correlated with bacterial communities in the present study. Further analysis revealed that sampling time could only explain a smaller variation (0.33%) in the total bacterial community. In contrast, fertilizer regime can explain a greater variation (7.43%) than sampling time and soil properties. Moreover, only fertilizer regime can significantly (*P* = 0.005) explain the variation in bacterial community structure.

In conclusion, fertilizer practice and seasonal changes can affect soil properties, microbial biomass, and bacterial community structure. We observed a significant and distinct seasonal shift for bacterial community while the fertilizer type also lead to a significant but much smaller variation in bacterial community structure. The treatment NPKMOI significantly increased the microbial biomass and biodiversity and may be more suitable for sustainable crop production.

Further study should pay more attention to the impact of the changes of bacterial community induced by fertilization and seasonal fluctuations on soil functionality.

## Supporting Information

Figure S1 **Rarefaction curves of bacterial communities for June (A) and October (B) based on the number of observed OTUs at 3% distance calculated from the randomly selected 10,000 pooled sequences of each treatment.** Error bars indicate 95% confidence intervals.
(TIF)

Table S1 **Soil available K of all samples from different fertilizer regimes both in June and October.**
(DOCX)

Table S2 **Soil microbial biomass C (MBC) and microbial biomass N (MBN) of all samples from different fertilizer regimes both in June and October.**
(DOCX)

Table S3 **Relative average abundances of phyla across all soils and soils grouped into fertilizer regime and sampling time categories, respectively (values represent % of total non-redundant sequences).** Asterisks mean values less than 0.01.
(DOCX)

Table S4 **Effects of fertilizer regime, sample time and the interaction between them on the abundant phyla (relative abundance >1%).**
(DOCX)

Table S5 **Effects of fertilizer regime, sample time and the interaction between them on the OTUs, coverage, richness and diversity calculated using a random 4,000 sequences per sample.**
(DOCX)
