# Supplementary figures and images for: Responses of Bacterial Communities in Arable Soils in a Rice-Wheat Cropping System to Different Fertilizer Regimes and Sampling Times
Source: PLoS One. 2014 Jan 20;9(1):e85301. doi: 10.1371/journal.pone.0085301 (PMC3896389; doi:10.1371/journal.pone.0085301)

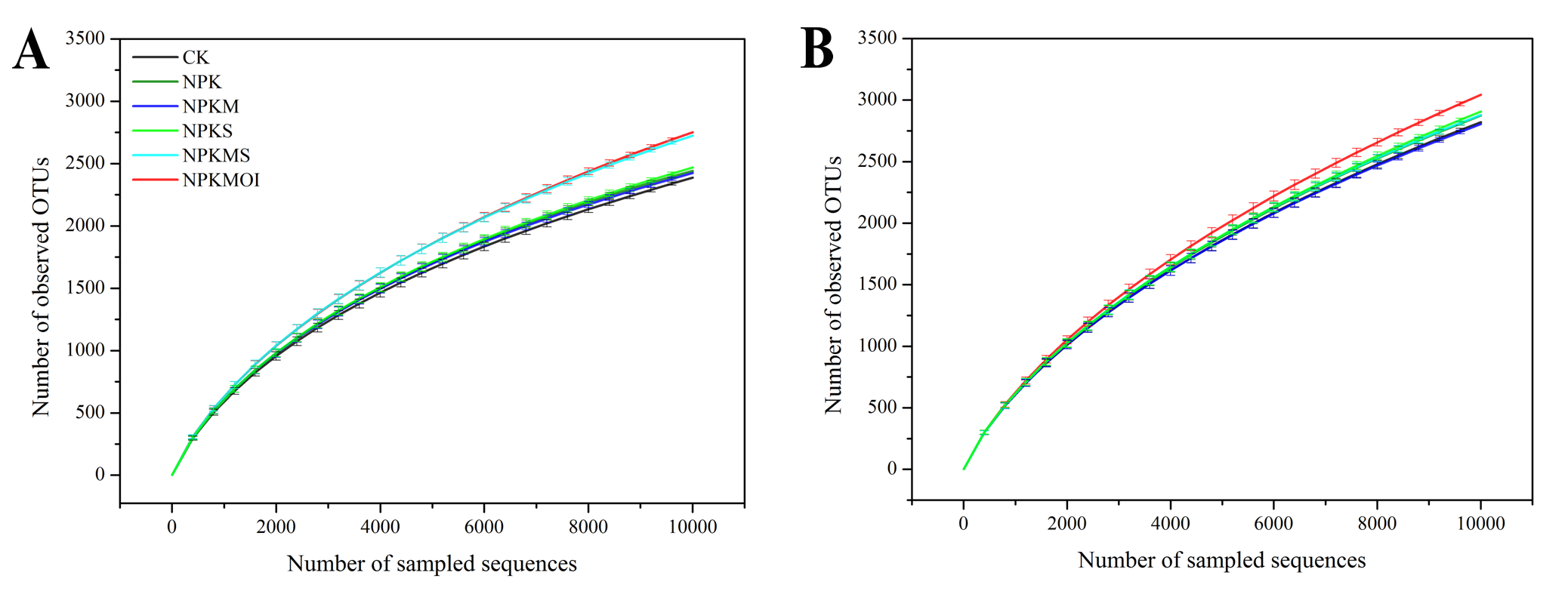

Supplement: Figure S1 — Rarefaction curves of bacterial communities for June (A) and October (B) based on the number of observed OTUs at 3% distance calculated from the randomly selected 10,000 pooled sequences of each treatment. Error bars indicate 95% confidence intervals. (TIF) [file pone.0085301.s001.tif]
