# Supplementary material for: Responses of Bacterial Communities in Arable Soils in a Rice-Wheat Cropping System to Different Fertilizer Regimes and Sampling Times
Source: PLoS One. 2014 Jan 20;9(1):e85301. doi: 10.1371/journal.pone.0085301 (PMC3896389; doi:10.1371/journal.pone.0085301)
Supplement: Table S1 — Soil available K of all samples from different fertilizer regimes both in June and October. (DOCX) [file pone.0085301.s002.docx]

Table S1. Soil available K of all samples from different fertilizer regimes both in June and October

| Fertilizer regime^§^ (FR) | Available K | |
| --- | --- | --- |
|  | June | October |
| CK | 71.39 ± 2.74 c | 42.69 ± 3.12 b |
| NPK | 83.54 ± 5.81 bc | 45.53 ± 2.32 ab |
| NPKM | 85.44 ± 6.24 bc | 46.78 ± 4.27 ab |
| NPKS | 100.35 ± 16.16 ab | 52.30 ± 3.13 ab |
| NPKMS | 92.13 ± 5.76 bc | 49.31 ± 4.46 ab |
| NPKMOI | 118.44 ± 12.25 a | 54.64 ± 5.73 a |

Values are means ± standard deviation (n=3).

Different lowercase letters in column indicate significant differences (*P* < 0.05) between different fertilizer regimes according to Turkey’s HSD test.

^§^Fertilizer regimes as described in Table 1.
