# Supplementary material for: Responses of Bacterial Communities in Arable Soils in a Rice-Wheat Cropping System to Different Fertilizer Regimes and Sampling Times
Source: PLoS One. 2014 Jan 20;9(1):e85301. doi: 10.1371/journal.pone.0085301 (PMC3896389; doi:10.1371/journal.pone.0085301)
Supplement: Table S2 — Soil microbial biomass C (MBC) and microbial biomass N (MBN) of all samples from different fertilizer regimes both in June and October. (DOCX) [file pone.0085301.s003.docx]

Table S2. Soil microbial biomass C (MBC) and microbial biomass N (MBN) of all samples from different fertilizer regimes both in June and October

| Fertilizer regime^§^ (FR) | MBC | |  | MBN |  |
| --- | --- | --- | --- | --- | --- |
|  | June | October |  | June | October |
| CK | 358.67 ± 93.32 c | 262.85 ± 62.19 b |  | 21.39 ± 6.78 d | 19.19 ± 6.91 b |
| NPK | 460.64 ± 38.76 ab | 314.87 ± 49.54 ab |  | 28.60 ± 2.43 cd | 28.12 ± 2.90 ab |
| NPKM | 514.30 ± 13.66 a | 343.17 ± 61.06 ab |  | 36.98 ± 3.84 abc | 30.43 ± 8.88 ab |
| NPKS | 470.96 ± 96.25 ab | 325.04 ± 93.96 ab |  | 29.11 ± 3.07 bcd | 31.34 ± 2.25 ab |
| NPKMS | 528.81 ± 67.50 a | 366.55 ± 51.55 ab |  | 38.85 ± 4.51 ab | 33.16 ± 5.29 a |
| NPKMOI | 540.85 ± 50.40 a | 401.54 ± 48.10 a |  | 41.67 ± 4.46 a | 36.03 ± 3.94 a |

Values are means ± standard deviation (n=4).

Different lowercase letters in column indicate significant differences (*P* < 0.05) between different fertilizer regimes according to Turkey’s HSD test.

^§^Fertilizer regimes as described in Table 1.
