# Supplementary material for: Responses of Bacterial Communities in Arable Soils in a Rice-Wheat Cropping System to Different Fertilizer Regimes and Sampling Times
Source: PLoS One. 2014 Jan 20;9(1):e85301. doi: 10.1371/journal.pone.0085301 (PMC3896389; doi:10.1371/journal.pone.0085301)
Supplement: Table S3 — Relative average abundances of phyla across all soils and soils grouped into fertilizer regime and sampling time categories, respectively (values represent % of total non-redundant sequences). Asterisks mean values less than 0.01. (DOCX) [file pone.0085301.s004.docx]

Table S3. Relative average abundances of phyla across all soils and soils grouped into fertilizer regime and sampling time categories, respectively (values represent % of total non-redundant sequences). Asterisks mean values less than 0.01.

| Phylum | All | June | October | CK | NPK | NPKM | NPKS | NPKMS | NPKMOI |
| --- | --- | --- | --- | --- | --- | --- | --- | --- | --- |
| *Proteobacteria* | 33.81 | 34.28 | 33.33 | 32.82 | 32.81 | 33.48 | 35.04 | 34.00 | 34.90 |
| *Acidobacteria* | 16.25 | 17.14 | 15.37 | 17.38 | 15.94 | 16.88 | 17.00 | 15.45 | 15.23 |
| *Chloroflexi* | 10.52 | 8.66 | 12.37 | 10.72 | 11.01 | 10.04 | 9.42 | 11.13 | 10.15 |
| *Bacteroidetes* | 7.69 | 9.20 | 6.19 | 7.01 | 8.02 | 8.23 | 7.59 | 7.79 | 7.55 |
| *Gemmatimonadetes* | 2.41 | 2.32 | 2.50 | 2.20 | 2.71 | 2.35 | 2.33 | 2.11 | 2.57 |
| *Actinobacteria* | 2.39 | 2.52 | 2.26 | 2.22 | 2.31 | 1.85 | 2.06 | 2.55 | 3.36 |
| *Verrucomicrobia* | 1.53 | 1.15 | 1.90 | 1.62 | 1.56 | 1.65 | 1.54 | 1.48 | 1.32 |
| *Nitrospira* | 1.35 | 1.67 | 1.03 | 1.27 | 1.51 | 1.25 | 1.49 | 1.41 | 1.20 |
| *WS3* | 1.13 | 0.94 | 1.32 | 1.26 | 1.21 | 1.33 | 1.17 | 1.01 | 0.86 |
| *Firmicutes* | 0.89 | 1.08 | 0.70 | 0.95 | 0.86 | 0.85 | 0.88 | 0.83 | 1.00 |
| *Armatimonadetes* | 0.70 | 0.74 | 0.67 | 0.66 | 0.69 | 0.65 | 0.70 | 0.75 | 0.71 |
| *TM7* | 0.54 | 0.70 | 0.37 | 0.19 | 0.40 | 0.36 | 0.42 | 0.42 | 1.41 |
| *Planctomycetes* | 0.50 | 0.49 | 0.50 | 0.53 | 0.48 | 0.45 | 0.44 | 0.55 | 0.54 |
| *Chlorobi* | 0.48 | 0.47 | 0.49 | 0.36 | 0.57 | 0.35 | 0.49 | 0.66 | 0.45 |
| *OD1* | 0.40 | 0.31 | 0.50 | 0.30 | 0.39 | 0.38 | 0.37 | 0.39 | 0.54 |
| *OP11* | 0.14 | 0.11 | 0.17 | 0.14 | 0.13 | 0.13 | 0.13 | 0.09 | 0.23 |
| *Spirochaetes* | 0.09 | 0.04 | 0.13 | 0.08 | 0.11 | 0.08 | 0.06 | 0.07 | 0.12 |
| *BRC1* | 0.08 | 0.08 | 0.07 | 0.09 | 0.11 | 0.07 | 0.07 | 0.04 | 0.07 |
| *Aquificae* | 0.05 | 0.09 | 0.00 | 0.08 | 0.05 | 0.03 | 0.05 | 0.05 | 0.03 |
| *Fibrobacteres* | 0.02 | 0.03 | 0.02 | 0.02 | 0.02 | 0.03 | 0.01 | 0.04 | 0.01 |
| *Lentisphaerae* | 0.01 | 0.00 | 0.01 | 0.01 | 0.01 | 0.00 | 0.02 | 0.01 | 0.00 |
| *Deinococcus-Thermus* | 0.00* | 0.00 | 0.00 | 0.00 | 0.00 | 0.00 | 0.00 | 0.00 | 0.00 |
| *SR1* | 0.00 | 0.00 | 0.00 | 0.00 | 0.00 | 0.00 | 0.00 | 0.00 | 0.00 |
| *Elusimicrobia* | 0.00 | 0.00 | 0.00 | 0.00 | 0.00 | 0.00 | 0.00 | 0.00 | 0.00 |
| Unclassified bacteria | 19.04 | 17.95 | 20.12 | 20.09 | 19.11 | 19.55 | 18.71 | 19.17 | 17.75 |
