# Supplementary material for: Responses of Bacterial Communities in Arable Soils in a Rice-Wheat Cropping System to Different Fertilizer Regimes and Sampling Times
Source: PLoS One. 2014 Jan 20;9(1):e85301. doi: 10.1371/journal.pone.0085301 (PMC3896389; doi:10.1371/journal.pone.0085301)
Supplement: Table S4 — Effects of fertilizer regime, sample time and the interaction between them on the abundant phyla (relative abundance >1%). (DOCX) [file pone.0085301.s005.docx]

Table S4. Effects of fertilizer regime, sample time and the interaction between them on the abundant phyla (relative abundance > 1%).

|  | *Proteobacteria* | *Acidobacteria* | *Chloroflexi* | *Bacteroidetes* | *Actinobacteria* | *Gemmatimonadetes* | *Verrucomicrobia* | *Nitrospira* |
| --- | --- | --- | --- | --- | --- | --- | --- | --- |
| Fertilizer Regime^§^(FR) |  |  |  |  |  |  |  |  |
| CK | 32.8 ± 1.6 a* | 17.4 ± 1.8 a | 10.7 ± 2.6 a | 7.0 ± 1.6 b | 2.2 ± 0.6 a | 2.2 ± 0.2 b | 1.6 ± 0.6 a | 1.3 ± 0.4 a* |
| NPK | 32.6 ± 2.0 a | 15.6 ± 1.6 bc | 11.6 ± 2.9 a | 8.0 ± 1.4 ab | 2.3 ± 0.5 a | 2.9 ± 0.4 a | 1.5 ± 0.6 a | 1.5 ± 0.4 a |
| NPKM | 33.5 ± 1.0 a | 16.9 ± 1.1 ab | 10.0 ± 1.8 a | 8.2 ± 1.3 a | 1.9 ± 0.3 a | 2.4 ± 0.3 ab | 1.6 ± 0.7 a | 1.3 ± 0.4 a |
| NPKS | 35.0 ± 1.8 a | 17.0 ± 0.9 ab | 9.4 ± 1.7 a | 7.6 ± 1.9 ab | 2.1 ± 0.3 a | 2.3 ± 0.4 b | 1.5 ± 0.7 a | 1.5 ± 0.4 a |
| NPKMS | 34.0 ± 1.8 a | 15.5 ± 1.5 bc | 11.1 ± 3.6 a | 7.8 ± 1.9 ab | 2.6 ± 1.6 a | 2.1 ± 0.2 b | 1.5 ± 0.7 a | 1.4 ± 0.4 a |
| NPKMOI | 34.9 ± 1.8 a | 15.2 ± 0.7 c | 10.1 ± 3.0 a | 7.6 ± 2.3 ab | 3.4 ± 1.7 a | 2.6 ± 0.5 ab | 1.3 ± 0.4 a | 1.2 ± 0.4 a |
| Sample Time (ST) |  |  |  |  |  |  |  |  |
| June | 34.3 ± 1.5 a | 17.1 ± 1.3 a | 8.7 ± 1.1 b | 9.2 ± 0.7 a | 2.5 ± 1.4 a | 2.3 ± 0.3 a | 1.2 ± 0.3 b | 1.7 ± 0.2 a |
| October | 33.3 ± 2.0 a | 15.4 ± 1.2 b | 12.4 ± 2.3 a | 6.2 ± 0.8 b | 2.3 ± 0.5 a | 2.5 ± 0.5 a | 1.9 ± 0.6 a | 1.0 ± 0.2 b |
| ANOVA *P*-values |  |  |  |  |  |  |  |  |
| FR | 0.025 | 0.001 | NS | 0.042 | NS | 0.003 | NS | 0.050 |
| ST | NS | < 0.001 | < 0.001 | < 0.001 | NS | NS | < 0.001 | < 0.001 |
| FR × ST | NS | NS | NS | NS | NS | NS | NS | NS |

Values are means ± standard deviation (n=6 or n=18).

NS: not significant (*P* > 0.05).

* Although fertilizer regime was a significant factor in the ANOVA, means comparison tests did not indicate any significant differences among fertilizer regimes.

Means followed by the same letter for a given factor are not significantly different (*P* < 0.05; Turkey’s HSD test where there are more than two treatment levels).

^§^Fertilizer regimes as described in Table 1.
