# Supplementary material for: Responses of Bacterial Communities in Arable Soils in a Rice-Wheat Cropping System to Different Fertilizer Regimes and Sampling Times
Source: PLoS One. 2014 Jan 20;9(1):e85301. doi: 10.1371/journal.pone.0085301 (PMC3896389; doi:10.1371/journal.pone.0085301)
Supplement: Table S5 — Effects of fertilizer regime, sample time and the interaction between them on the OTUs, coverage, richness and diversity calculated using a random 4,000 sequences per sample. (DOCX) [file pone.0085301.s006.docx]

Table S5. Effects of fertilizer regime, sample time and the interaction between them on the OTUs, coverage, richness and diversity calculated using a random 4,000 sequences per sample.

|  | OTUs^1^ | Coverage^2^ (%) | Richness^3^ | | Diversity^4^ | |
| --- | --- | --- | --- | --- | --- | --- |
|  |  |  | ACE | Chao1 | Shannon | Simpson |
| Fertilizer Regime^§^ (FR) |  |  |  |  |  |  |
| CK | 1455 ± 65 a | 0.79 ± 0.02 a | 3993 ± 509 a | 2784 ± 264 a | 6.63 ± 0.04 a | 0.0030 ± 0.0005 a |
| NPK | 1466 ± 101 a | 0.79 ± 0.03 a | 4025 ± 868 a | 2814 ± 381 a | 6.64 ± 0.05 a | 0.0030 ± 0.0005 a |
| NPKM | 1476 ± 91 a | 0.79 ± 0.03 a | 4217 ± 1020 a | 2905 ± 519 a | 6.66 ± 0.04 a | 0.0028 ± 0.0003 a |
| NPKS | 1473 ± 96 a | 0.79 ± 0.03 a | 4100 ± 947 a | 2901 ± 454 a | 6.67 ± 0.05 a | 0.0027 ± 0.0004 a |
| NPKMS | 1529 ± 104 a | 0.78 ± 0.03 a | 4319 ± 1084 a | 2968 ± 480 a | 6.70 ± 0.09 a | 0.0028 ± 0.0007 a |
| NPKMOI | 1565 ± 121 a | 0.77 ± 0.03 a | 4621 ± 748 a | 3156 ± 407 a | 6.73 ± 0.12 a | 0.0027 ± 0.0005 a |
| Sample Time (ST) |  |  |  |  |  |  |
| June | 1427 ± 97 b | 0.80 ± 0.02 a | 3527 ± 594 b | 2601 ± 313 b | 6.66 ± 0.09 a | 0.0026 ± 0.0003 b |
| October | 1560 ± 37 a | 0.76 ± 0.01 b | 4898 ± 356 a | 3241 ± 189 a | 6.69 ± 0.04 a | 0.0031 ± 0.0005 a |
| ANOVA *P*-values |  |  |  |  |  |  |
| FR | NS | NS | NS | NS | NS | NS |
| ST | < 0.001 | < 0.001 | < 0.001 | < 0.001 | NS | 0.001 |
| FR × ST | NS | NS | NS | NS | NS | NS |

Values are means ± standard deviation (n=6 or n=18).

NS: not significant (*P* > 0.05).

Means followed by the same letter for a given factor are not significantly different (*P* < 0.05; Turkey’s HSD test where there are more than two treatment levels).

^1^OTUs: operational taxonomic units (97% similarity).

^2^Coverage: Good’s non-parametric coverage estimator.

^3^Based on Chao1 and abundance-based coverage estimator (ACE) richness indices.

^4^Based on Shannon and Simpson diversity indices.

^§^Fertilizer regimes as described in Table 1.
